# Biosafety Test for Plant Growth-Promoting Bacteria: Proposed Environmental and Human Safety Index (EHSI) Protocol

**DOI:** 10.3389/fmicb.2015.01514

**Published:** 2016-01-07

**Authors:** Juan I. Vílchez, Alfonso Navas, Jesús González-López, Susana C. Arcos, Maximino Manzanera

**Affiliations:** ^1^Institute for Water Research and Department of Microbiology, University of GranadaGranada, Spain; ^2^Biodiversidad y Biologia Evolutiva, Museo Nacional de Ciencias Naturales, Consejo Superior de Investigaciones CientíficasMadrid, Spain

**Keywords:** plant-growth-promoting-bacteria, inoculants, intentional-release, biosafety-index, ecotoxicity

## Abstract

Plant growth-promoting bacteria (PGPB) colonize plants and enhance their growth by different mechanisms. Some of these microorganisms may represent a potential threat to human, animal or plant health; however, their use might be approved in parts of Europe if they have been recommended as plant growth enhancers. The current regulatory framework has resulted in a fragmented, contradictory system, and there is an urgent need to establish harmonized protocols for the predictability, efficiency, consistency and especially the safety of PGPB for human and animal health and for the environment. In response to current efforts to update biosafety policies and provide alternative methods to replace the use of vertebrate animals, we propose a panel of tests and an evaluation system to reliably determine the biosafety of bacterial strains used as PGPB. Based on the results of different tests, we propose a scoring system to evaluate the safety of candidates for PGPB within the limitations of the assays used.

## Introduction

Bacteria that are beneficial for plant growth have been used since the 1970s to increase crop production (Rovira et al., 1974; Vilchez and Manzanera, 2011). These microoganisms affect plants in different ways that include increasing nutrient bioavailability and bioassimilation, reducing the pathogenic effects of soil plant pathogens, producing substances that enhance plant growth and removing from the soil detrimental molecules such as toxic compounds that can impair plant growth (Glick, 1995; Kuiper et al., 2001; Vassilev et al., 2006; Adesemoye et al., 2008). These microorganisms are termed plant growth-promoting bacteria (PGPB), and their use represents an alternative to organic and inorganic fertilizers, pesticides and transgenic plants. In addition, PGPB can overcome the detrimental effects of environmental stresses in soils. Among these sources of stress are high salt concentration (Mayak et al., 1999; Nadeem et al., 2010), pollution by heavy metals and other inorganic compounds (Dimkpa et al., 2009; Ma et al., 2011) or by organic pollutants such as polycyclic aromatic hydrocarbons, and drought. Thus PGPB are potentially important as an aid to reclaiming farmland that was not previously cultivable for feed or food.

Plant growth-promoting bacteria can be found associated to plant roots, shoots, and leaves, or in the fruits or seeds (Laca et al., 2006). These associations suggest a very close relationship or symbiosis between plants and microorganisms. In this sense, the safety of handling and processing inoculated plants must be ensured, not only to protect humans but also to protect the environment, just as with organic and inorganic fertilizers.

The European Parliament and the Council of the European Union are working to produce new regulations for 2017. Currently, Regulation (EC) No 2003/2003 aims to regulate the correct use of materials intended mainly to provide nutrients to plants, regardless of whether microorganisms or other types of products are involved. Regulation (EC) No 1107/2009 recognizes explicitly that “plant protection products may involve risks and hazards for humans, animals and the environment, especially if placed on the market without having been officially tested and authorized and if incorrectly used.” This regulation further establishes that “in the interest of predictability, efficiency and consistency, criteria procedures and conditions for the authorization of plant protection products should be harmonized, account being taken of general principles of protection of human and animal health and the environment.” Regulatory harmonization, however, cannot rely on animal tests as noted in “the development of non-animal test methods should be promoted in order to produce safety data relevant to human and to replace animal studies currently in use.” Moreover, Directive 2010/63/EU specifies that “minimized animal testing and tests on vertebrates should be undertaken as a last resort.”

In light of the need to comply with current regulations aimed to ensure human and environmental safety, we developed a set of biosafety tests for PGPB that assess the potential impacts of the products released by these microorganisms on microbial metabolism (Microtox^®^ testing in *Vibrio fischeri* ATCC 49387), microbial viability (*Escherichia coli* MC4100 sensitivity test), the survival and viability of soil nematodes (*Caenorhabditis elegans* bioassay) and earthworms (*Eisenia foetida* bioassay). An additional aim of our tests was to assess potential harms to the organisms at the second trophic level of the soil cycle (primary consumers). We also undertook assays to assess the effect on organisms from the third trophic level (secondary consumers), including the arthropods *Adalia bipunctata* (neuropteran) and *Chrysoperla carnea* (colleoptera). These are two of the most abundant predatory arthropod species in nature, and both are widely used in the biological control of pests. We also tested whether PGPB could cause harm to organisms that characterize fragile, wet ecosystems, which are considered important reservoirs of biodiversity. Accordingly, we carried out bioassays with *Daphnia magna*. Finally, with a view to developing an alternative test method that does not require further experimentation in mammals (Medina et al., 2004; OECD, 2004, 2008; Onorati and Mecozzi, 2004; Vassilev et al., 2006; Navas et al., 2007; Alvarez-Alfageme et al., 2011) or other vertebrates, we performed bioassays in laboratory mice (*Mus musculus*).

In the tests reported here we also considered whether, according to EC Regulation No 1107/2009 the microorganisms “present a clear benefit for plant production.”

The results of all tests were translated into a scoring system to generate what we termed the *environmental and human safety index* (EHSI), which we propose as a system to evaluate the safety of recommended uses of specific PGPB.

In this work we piloted the EHSI to test the safety of a minimum of 10^8^ cells of *Pseudomonas putida* KT2440 and *Burkholderia cepacia* CC-A174, since both *P. putida* and *B. cepacia* strains have been proposed as PGPB in the literature. The former strain is generally considered to be mostly innocuous, whereas *B. cepacia* CC-A174 is classified as a risk group 2 strain (Grimes and Mount, 1984; Govan et al., 1996; Nacamulli et al., 1997; Sundh et al., 2011). In addition other eight strains including well known characterized PGPB strains, and well known pathogenic strains have been included in the study to validate this index.

## Materials and Methods

### Strains

The bacterial strains we used were *Pseudomonas putida* KT2440 as Risk Group 1 representative, *Burkholderia cepacia* CC-Al74 as Risk Group 2 and proposed as PGPR, *Escherichia coli* MC4100 used as microbial model in sensitivity and microbial metabolism assays, *Escherichia coli* OP50 used to feed *C. elegans* (Brenner, 1974), *Vibrio fischeri* ATCC 49387 is commonly used as bioluminescent strain in MicroTox assays (Onorati and Mecozzi, 2004; Perry et al., 2005), and *Pseudomonas aeruginosa* PA14 as Risk Group 2 representative for some animal tests (Tan et al., 1999). In addition strains *Rhizobium legominosarum* IABRL05, *Pseudomonas fluorescens* IABPF05, *Bacillus subtilis* IABBS05, and *Azotobacter vinelandii* IABAV02 provided by Aplicaciones Biotecnológicas S.L. (Spain) as PGPB; and *Serratia marcescens* 615 (Almaghrabi et al., 2013), *Serratia entomophila* A1 (Johnson et al., 2001), *Serratia proteamaculans* 28151 (Bai et al., 2002), and *P. aeruginosa* PA14 as potential PGPB and pathogenic strains were included in this study to validate the Index. Unless otherwise specified, bacteria were grown at 30°C in trypticase soy agar (TSA) or broth. The *C. elegans* wild-type Bristol strain N2 we used was provided by the Laboratory of Nematology, National Museum of Nature Sciences-CSIC (Madrid, Spain) (originally provided by Genetics Center, Minneapolis, MN). *Adalia bipunctata* and *Chrysoperla carnea* were obtained from ControlBio Co. (Almería, Spain; Ref. CBi K04884 and CBi K04280 respectively). Both *A. bipunctata* and *C. carnea* were grown in 15 × 15 × 25 cm terrarium and were fed with frozen Mediterranean fruit fly (*Ceratitis capitata*) eggs (Dept. of Parasitology, University of Granada, Spain) (∼1,000 eggs every 3 days). Earthworms (*E. foetida*) were obtained from Lombriventa (Gerona, Spain).

### *Escherichia coli* MC4100 Sensitivity and Microbial Metabolism Assays

Sensitivity assays in *Escherichia coli* MC4100 were performed according to Small et al. (1994) and others (Peters et al., 2003) with some modifications. Filtered sterilized supernatants (0.5 mL) from stationary-phase cultures of the PGPB candidates were mixed with 0.5 mL of an *E. coli* MC4100 suspension in M9 sterile buffer containing approximately 10^8^–10^9^ cells collected from a mid-log phase culture. The mixtures were incubated for 1.5 h at room temperature, then serial dilutions from the *E. coli* MC4100 suspension were mixed with 0.5 mL sterile supernatant. Samples containing *E. coli* suspensions were also mixed with 0.5 mL TSB as a negative control. The mixtures were plated on TSA plates to estimate CFU⋅mL^-1^.

Light emission by *V. fischeri* ATCC 49387 is finely tuned to the microorganism’s metabolism. To detect secondary metabolites with negative effects on cell metabolism independently of their lethal potential, experiments were done according to the manufacturer’s recommendations using 1-mL of the sterile supernatants noted above.

### Pathogenicity Bioassay Based on *Caenorhabditis elegans*

Bacterial killing of *C. elegans* was assayed as previously described by Darby and coworkers (Darby et al., 1999), with some modifications as described by Navas et al. (2007) and Ruiz-Díez et al. (2003). Briefly, each PGPB candidate was spread on five potato dextrose agar plates and incubated at 30°C for 24 h. Then each plate was seeded with five adult hermaphrodite individuals adding a total of 25 adults. The plates were incubated at 24°C, and nematodes were examined at 20× and 40× magnification and counted every 24 h thereafter for 7 days. In all cases we used *E. coli* strain OP50 as a control to estimate the natural death rate of the nematode, and *P. aeruginosa* PA14 as a control for the pathogenic strain.

### Ecotoxicity Tests in Green Lacewings (*Chrysoperla carnea*) and Ladybirds (*Adalia bipunctata*)

These bioassays were carried out according to Medina et al. (2004) and Alvarez-Alfageme et al. (2011) with slight modifications. A total of five cages were employed, including a single L1 larval stage insect per cage that was fed with *C. capitata* eggs combined with the bacterial strain of interest, which was preserved by drying with trehalose as a lyoprotectant (10% wt/vol trehalose lyophilized for 24 h according to Manzanera et al. (2004). The food was prepared by mixing approximately 1,000 eggs with 0.1 g lyophile (the stable formulation of the candidate PGPB dried in a 10% wt/vol solution of trehalose). The lyophile contained the test strain at 10^8^–10^9^ CFU⋅g-1, and the insects were fed three times per week at different times. Body length and weight and mortality under each condition were recorded at 0, 7, and 15 days. A negative control was included by adding eggs combined with an equivalent amount of sterile trehalose. All experiments were conducted in a climate chamber at 25 ± 1°C, 60 ± 5% relative humidity, and a 16-h photoperiod.

### Ecotoxicity Tests in Earthworms (*Eisenia foetida*)

Earthworm reproduction tests (*E. foetida*/*E. andrei*) were carried out according to OECD Test Guideline Test No. 222 (Neuhauser and Callahan, 1990; OECD, 2004) with slight modifications. Ten earthworms of the same generation (at least 1 month old and about 5.5–6 cm long) were placed in containers filled with 0.5 L of a mixture of vegetal substrate and sphagnum peat (3:1, wt/wt) and were kept at pH 6.7–7.4, 70% relative humidity and 20–25°C for 30–60 days in the dark. The earthworm were fed with moistened chickpea flour (5 g per week). Bacteria were added as a freeze-dried formulation with trehalose (10^8^–10^9^ CFU/g) (0.5 g per week). As a control for earthworm death rate, NaCl (2% wt/wt) was added to the substrate mixture as a dry powder. At each sampling time (30 and 60 days), length and weight of the initial individuals, clitellum formation, the number of ootheca and the number of juveniles were recorded.

### DaphToxKit^®^: *Daphnia magna* Toxicity Bioassay

The toxicity of bacterial extracts to *D. magna* (Cladocera) was assayed with DaphToxKit F^®^ (Microbiotests, BE) (Hernando et al., 2003) accordingly to ISO 6341 and OECD Guideline no. 211 (OECD, 2008). Tests were done in the dark at 20°C for 24 and 48 h, after which immobility was recorded. Twenty newly hatched animals (24 h old form ephippia) were transferred to a multiwell microplate system (10 mL/well; 5 animals/well) for each tested concentration of bacterial extract. The contents of each well were directly dissolved in test “freshwater” in the absence of cosolvents or vehicles. An individual was considered immobile when it did not swim (even if moving the antennae) during a 15-s observation period. Potassium dichromate was also tested as a reference to verify that our laboratory test conditions did not significantly affect the sensitivity of the test.

### Ethics Statement and Pathogenicity Test in Laboratory Mice (*Mus musculus* CD1)

Pathogenicity in an animal model was tested with a modification of the method of Stelma et al. (1987). Bacterial strains were grown for 24 h at 30°C in trypticase soy broth plus 0.6% yeast extract. Cultures were concentrated 10-fold by centrifugation and then suspended in 0.1% peptone. Five CD1 female mice (23–24 g) were injected intraperitoneally (i.p.) with 0.1 mL of the suspension, containing approximately 10^9^ cells. Individuals to be tested with the same bacterial strain were housed together in 4-L racks at 25°C, under a day-night light cycle and controlled ventilation. They were fed with sterile mouse chow and water. The mice were observed for 2 weeks, and body weight and the number of deaths were recorded at 7 and 14 days. Strains that killed 3 or more mice were considered to be pathogenic (assuming a natural death rate of no more than 20%). As a negative control we injected a 0.1% peptone solution i.p. With regard to animal care and laboratory use during the survival study, we indicate that a total group of 15 mice were employed over 2 weeks experimental procedure. Animals were inspected on a daily basis. Euthanasia of animals was applied to those individuals that showed a loss of 20–25% body weight, in case of maintained inappetance (no consumption of food for 24 h) or lack of response to gentle stimuli (moribund appearance). In addition all animals were subjected to euthanasia at the end of the experiment (2 weeks) to provide a humane endpoint using a commercial euthanasia solution (Euthanal) consisting of a mixture of sodium pentobarbital 390 mg with sodium phenytoin 50 mg/ml. To that end 0.22 mL/kg was IP administered (∼86 mg/kg sodium pentobarbital). In addition oral analgesia was used to relief mice of pain. To that end 60 mg/kg/day of ibuprofen was added to their dinking water. All animal experiments were performed in compliance with national and international regulations and were approved by the Ethical Review Board of the University of Granada under the project number P11-RNM-7844. The procedures employed complied with the National (“Real Decreto” 1201/2005 and 53/2013) and European (Directives 86/609/CEE and 53/2013) regulations.

### Bacterial Effects in Pepper (*Capsicum annuum*) Plants

Growth promotion of pepper plants inoculated with PGPB candidates was tested according to Mayak et al. (2001) with minor modifications. Pots (0.4 L) were filled with sterile vermiculite and vegetable substrate (50% vol/vol) and seeded with sterilized pepper seeds. When the seedlings reached 2 cm, they were inoculated with 40 mL of bacterial inoculum (10^8^–10^9^ CFU/mL) in M9 sterile saline solution. The plants were weekly irrigated with 40 mL sterile distilled water. Three seedlings per condition were sampled on days 7, 14, 21, and 33, and height, fresh weight, fully turgid weight and dry weight (DW) were recorded. As a negative control M9 buffer without the bacterial inoculum was used.

### Statistical Analyses

All tests were performed independently tree times. For statistical testing, analysis of variance (ANOVA) was used for each test with a significance level of *p* < 0.05. All analyses were done with STATISTICA v. 10.0 software (StatSoft Inc., Tulsa, OK, USA).

## Results

### Effect on Microbial Communities

The potential effect of PGPB candidates on microbial communities was assessed in two different areas: microbial viability (sensitivity assay) and microbial metabolism (bioluminescence assay). The premise is that secondary metabolites produced and released to the environment can be collected from the growth medium, most likely during the idiophase. Therefore we studied the effect on *E. coli* MC4100 cells of supernatants of cultures in the stationary phase of *P. putida* KT2440 and *B. cepacia* CC-A174. A 50% reduction in survival of *E. coli* MC4100 was found when bacteria were exposed to the supernatant from *B. cepacia* CC-A174. However, no statistically significant change in survival was observed when the supernatant from *P. putida* KT2440 cultures was used instead. These supernatants were also tested with *V. fischeri* to determine whether any change in metabolism was detectable as a change in light emission (Onorati and Mecozzi, 2004). The results were recorded as the effective concentration (EC50), defined as the concentration of supernatant that caused a 50% decrease in the light emitted by *V. fischeri*. Exposure to *P. putida* KT2440 supernatants resulted in a very high median EC50 (77.81% ± 2.02%), indicating little effect, whereas exposure to supernatants from *B. cepacia* CC-A174 resulted in a much lower EC50 (23.75% ± 2.31%), indicating a marked effect on bacterial metabolism. Lower concentrations of supernatant were required to reduce the *V. fischeri* bioluminescence, especially in comparison to fresh tryptic soy broth (TSB) medium, which had no effect on bioluminescence.

### Pathogenocity Bioassasy with *Caenorhabditis elegans, Chrysoperla carnea*, and *Adalia bipunctata*

**FIGURE 1:**
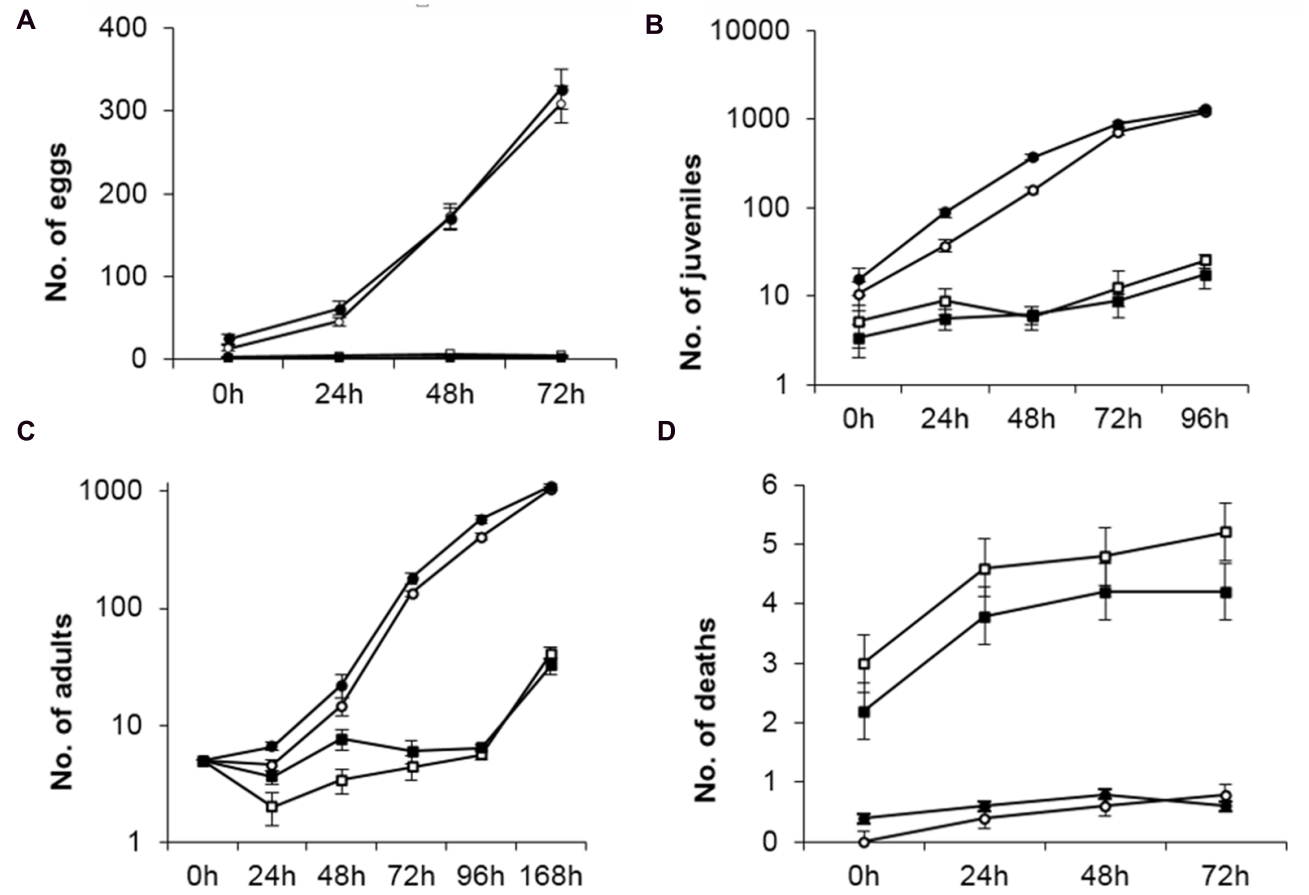
**Pathogenicity bioassay in *Caenorhabditis elegans*.** Time course of changes in number of eggs laid **(A)**, juveniles **(B)**, adults **(C)** and dead **(D)** organisms found after feeding *C. elegans* with *P. putida* KT2440, *B. cepacia* CC-A174, *E. coli* OP50 or *P. aeruginosa* are represented along time. The values shown are the mean and standard deviation of three measurements.

*Caenorhabditis elgans* is considered a reliable model for the study of the transmission and development of several diseases that occur in higher organisms, including humans. Our pathogenicity bioassays in nematodes were designed to evaluate the effect of bacterial strains on the number of eggs laid, number of juveniles, number of adults and death rate (Navas et al., 2007). When *C. elegans* was fed with *P. putida* KT2440 the numbers of eggs laid, juveniles, adults and individuals that died after 72 h were similar to those observed when the nematodes were fed with *E. coli* OP50, a well established non-pathogenic strain. However, feeding with *B. cepacia* CC-A174 resulted in significantly lower numbers of eggs, juveniles and adult worms (approx. half the numbers obtained with *E. coli* OP50). The numbers were similar to those we observed when *P. aeruginosa* PA14, a standard pathogenic control strain, was used as feed (**Figure 1**).

**FIGURE 2:**
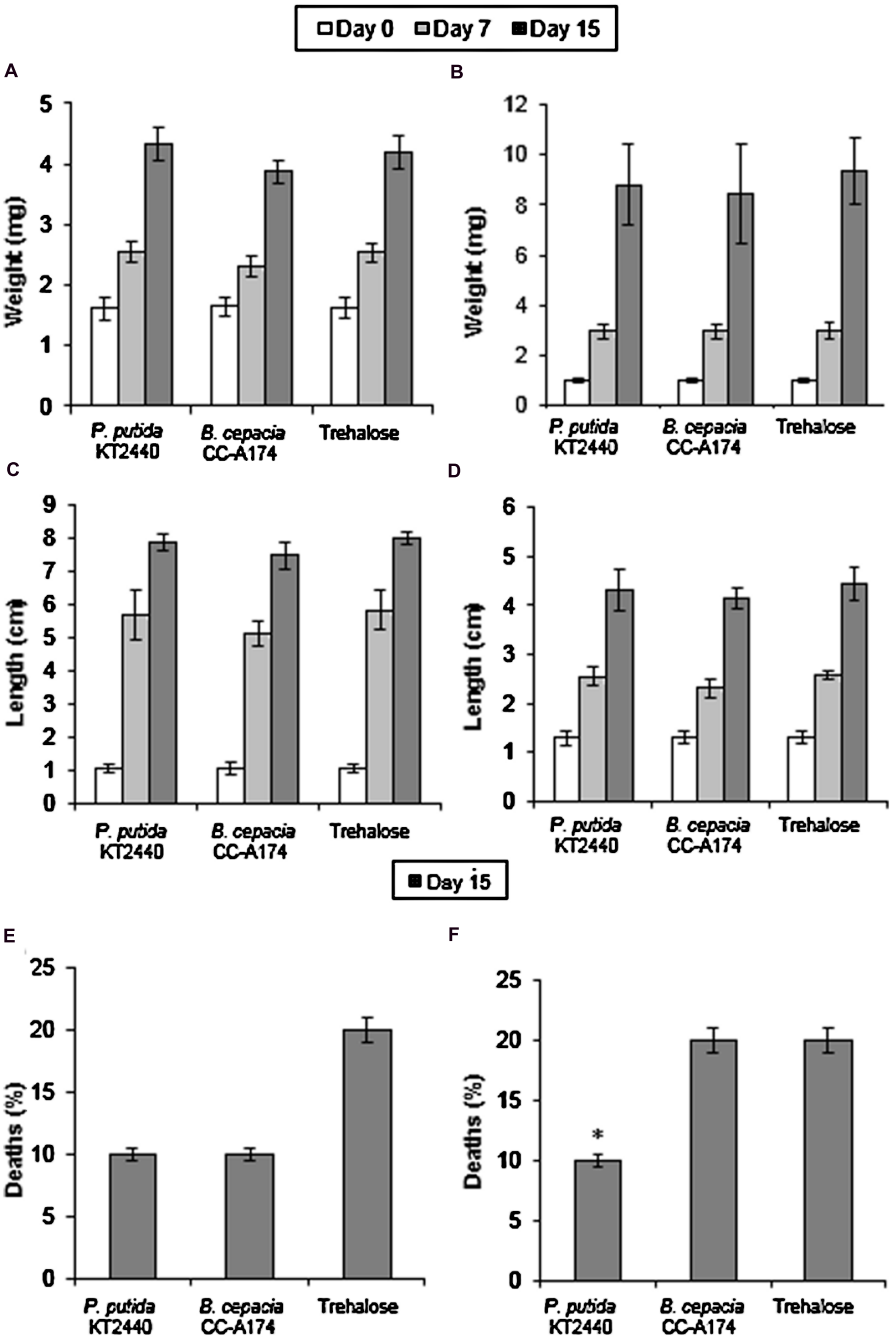
**Ecotoxicity tests in green lacewings (*Chrysoperla carnea*) and ladybirds (*Adalia bipunctata*).** Time course of changes in weight **(A,B)**, length **(C,D)** and mortality rates **(E,F)** of *C. carnea* and *A. bipunctata* after incubation with *P. putida* KT2440 or *B. cepacia* CC-A174, or after the addition of an equivalent amount of trehalose. Significant differences are indicated with an asterisk. The values shown are the mean and standard deviation of three measurements.

Apart from its potential effect on the nematode community in the environment, the addition of a PGPB candidate to soil or plants can potentially alter the abundance of non-pest herbivores, predators and parasitoids of pest species, or pollinators, and this in turn will affect the environmental equilibrium. Green lacewings (*Chrysoperla carnea*) and ladybirds (*Adalia bipunctata*) are insects that are considered good biological control agents which are beneficial for both natural and farm ecosystems, since both prey on most pest or phytopathogenic insects such as aphids and whiteflies as well as other arthropods. However, these organisms are fragile and may be affected by pesticides or bacterial infections. This makes it essential to test the impact of PGPB as part of any evaluation of an environmental safety index (or other instrument). We measured the changes in weight and length, and in the numbers of dead insects. To obtain a solid diet and ensure that bacteria were distributed evenly in the diet, these microorganisms were freeze-dried with trehalose as a lyoprotectant. When *C. carnea* and *A. bipunctata* were fed with *P. putida* KT2440 dried on trehalose, weight and length were similar to those of insects that were fed the trehalose control without bacteria. When they were fed with *B. cepac*ia CC-A174, weight and size were slightly lower than in the control group although the differences were not statistically significant. However, when *A. bipunctata* was fed with *B. cepacia* CC-A174 the death rate was 20% compared to 10% when they were fed with *P. putida* KT2440 (**Figure 2**). It should nevertheless be noted that mortality rates between 10 and 20% are within the normal range for this type of assay.

### Ecotoxicity Tests in Earthworms (*Eisenia foetida*)

**FIGURE 3:**
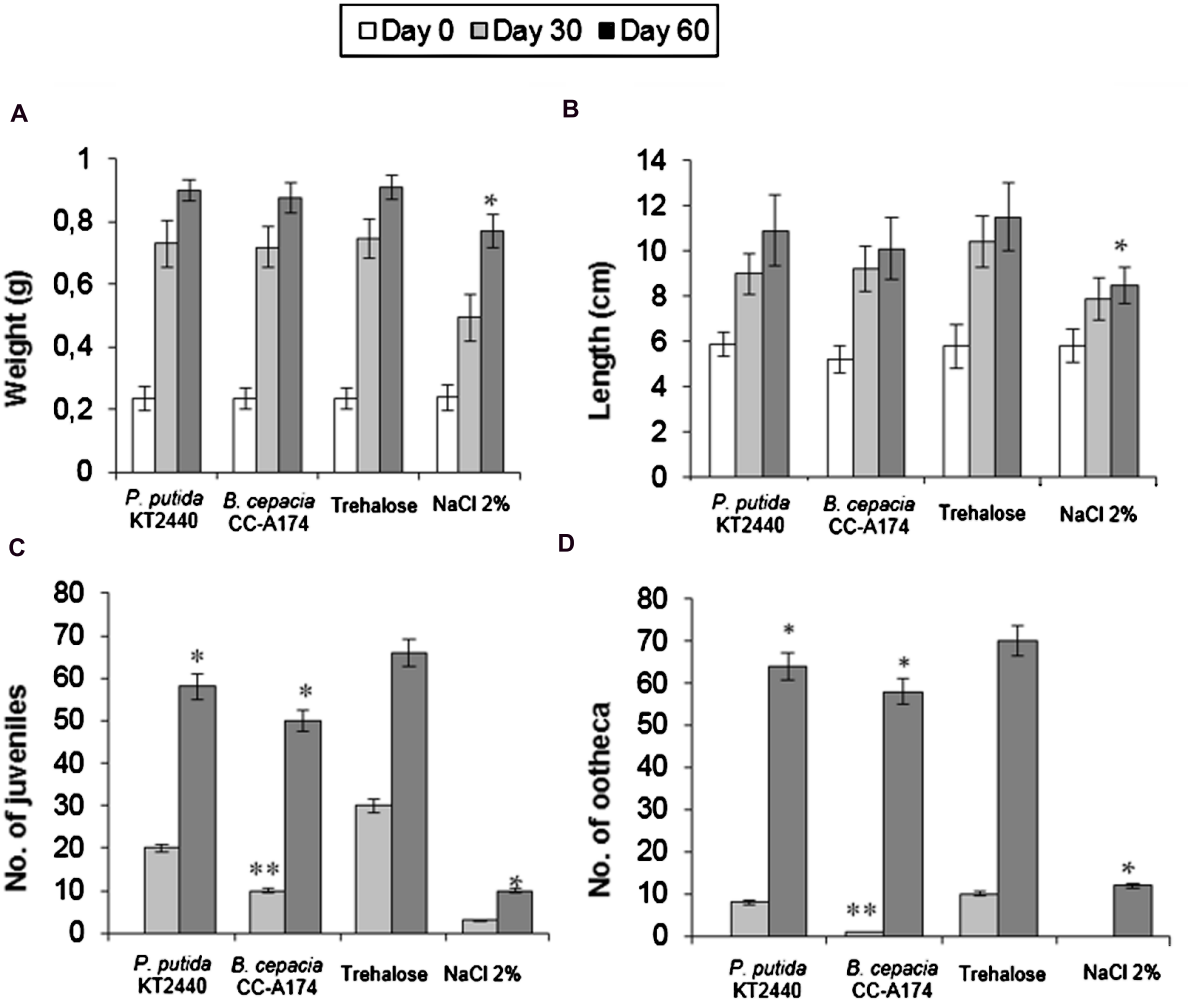
**Ecotoxicity tests in earthworms (*Eisenia foetida*).** Time course of changes in weight **(A)** length **(B)**, number of juveniles **(C)** and number of ootheca laid **(D)** by *Eisenia foetida* after incubation with *P. putida* KT2440 or *B. cepacia* CC-A174, or after the addition of an equivalent amount of trehalose or 2% NaCl. Significant differences are indicated with an asterisk. The values shown are the mean and standard deviation of three measurements.

Although published reports are available on the impact of chemicals (Nahmani et al., 2007) and specifically metals (Coeurdassier et al., 2007; Fernández et al., 2009; Sizmur and Hodson, 2009; Gomez-Eyles et al., 2011; Sizmur et al., 2011) on earthworms, few studies have focused on the impact of microorganisms in soil annelids. We therefore felt it was important to investigate the potential effects of PGPB on *E. foetida* development, which we measured as weight gain, length increase and reproductive success (number of juveniles and oothecas). These tests yielded similar values for weight gain (78%) and length increase (45%) with both *P. putida* KT2440 or *B. cepacia* CC-A174, and these values did not differ significantly from the increases in control earthworms not exposed to bacteria (**Figures 3A,B**). The addition of 2% NaCl resulted in much smaller increases in weight (69%) and length (32%). However, the addition of 10^9^ CFU of *P. putida* KT2440 or *B. cepacia* CC-A174 dried with trehalose to soils containing live *E. foetida* reduced the number of juveniles by 12.12% and 16.67% respectively compared to exposure to trehalose alone (**Figure 3C**). The number of egg cases (ootheca) was reduced by 8.57% after *P. putida* KT2440 was added, and by 17.14% after *B. cepacia* CC-A174 was added (**Figure 3D**). These results showed that exposure to either of these two bacterial strains had significant effects on *E. foetida* biocycles (development and reproduction) when they were exposed to a concentration of bacterial cells higher than that normally used for plant inoculation.

### DaphToxKit^®^: *Daphnia magna* Toxicity Bioassay

*Daphnia magna* is a useful marker to assess the impacts of introduced substances, including microorganisms, on aquatic ecosystems. Bioassays with DaphToxKit^®^ led to very low EC50 values (defined as the concentration of supernatant that killed 50% of *D. magna* individuals) after the addition of *P. putida* KT2440 (14.12% ± 1.78%) or *B. cepacia* CC-A174 (12.50% ± 0.5%), indicating that at low concentrations these strains could negatively affect *D. magna* mobility and survival. In assays with the carrier medium (TSB) used to prepare the suspensions of both strains, and with standard freshwater, we observed no effect on *D. magna* survival. The addition of both strains at the assayed concentration had an adverse effect on *D. magna* survival at certain concentrations.

### Plant Growth-Promoting Effect of Bacterial Strains

The addition of PGPB candidates to enhance plant growth should not have any detrimental effect on other plant species. To address this concern, we tested both bacterial strains in pepper plants (*Capsicun annuum*) as a model of widely cultivated and well-studied crop species.

**FIGURE 4:**
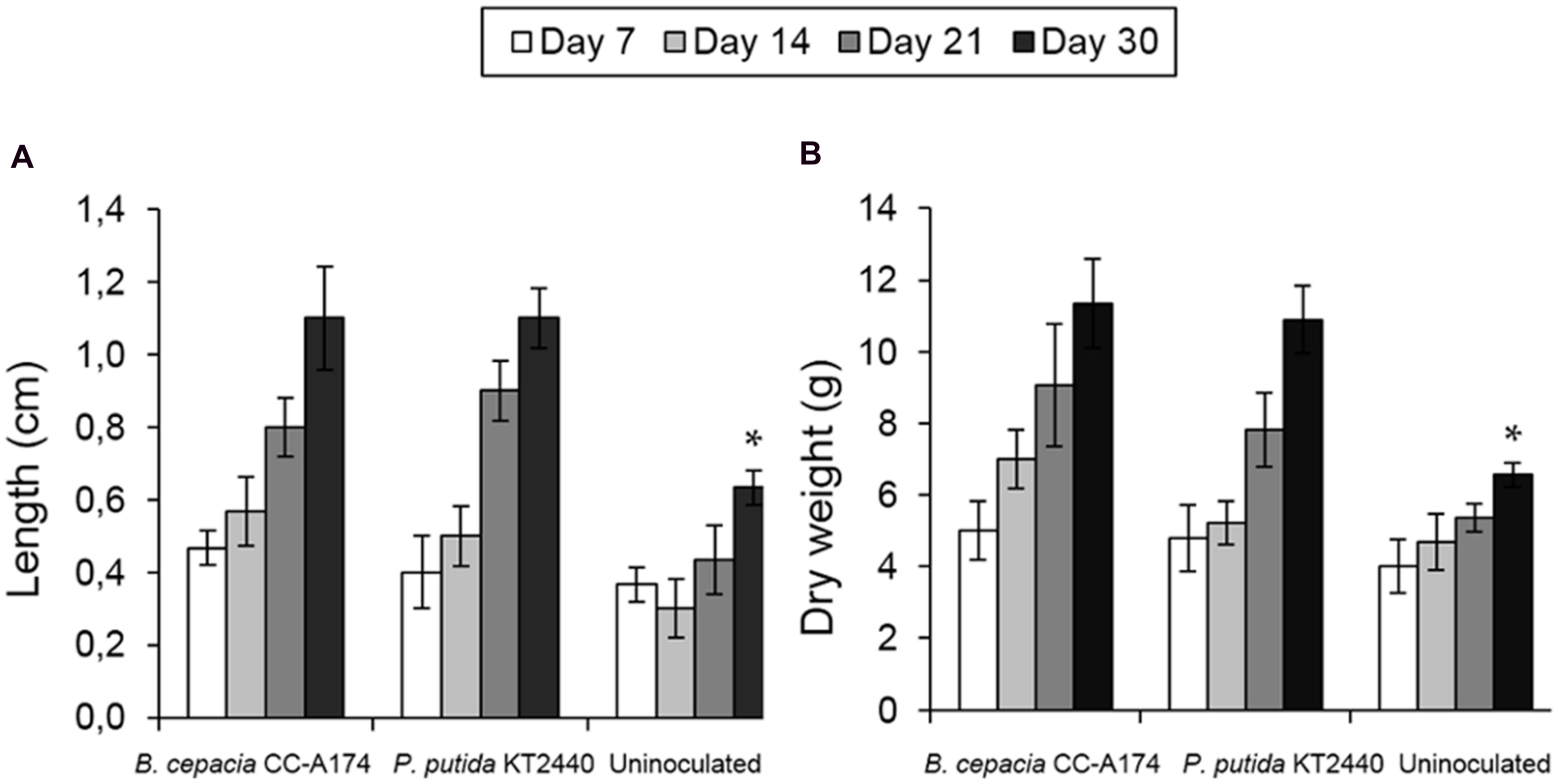
**Plant growth promotion by bacterial strains.** Plant shoot length **(A)** and plant dry weight **(B)** in uninoculated pepper plants (*Capsicum annuum*) and plants inoculated with *P. putida* KT2440 or *B. cepacia* CC-A174. Significant differences are indicated with an asterisk. The values shown are the mean and standard deviation of three measurements.

The addition of *P. putida* KT2440 or *B. cepacia* CC-A174 led to increased plant shoot length, larger root systems and increased dry and fresh weight compared to non-inoculated plants (**Figures 4A,B**). Both microorganisms promoted growth without any adverse effects.

### Pathogenicity Tests in Laboratory Mice (*Mus musculus* CD1)

**FIGURE 5:**
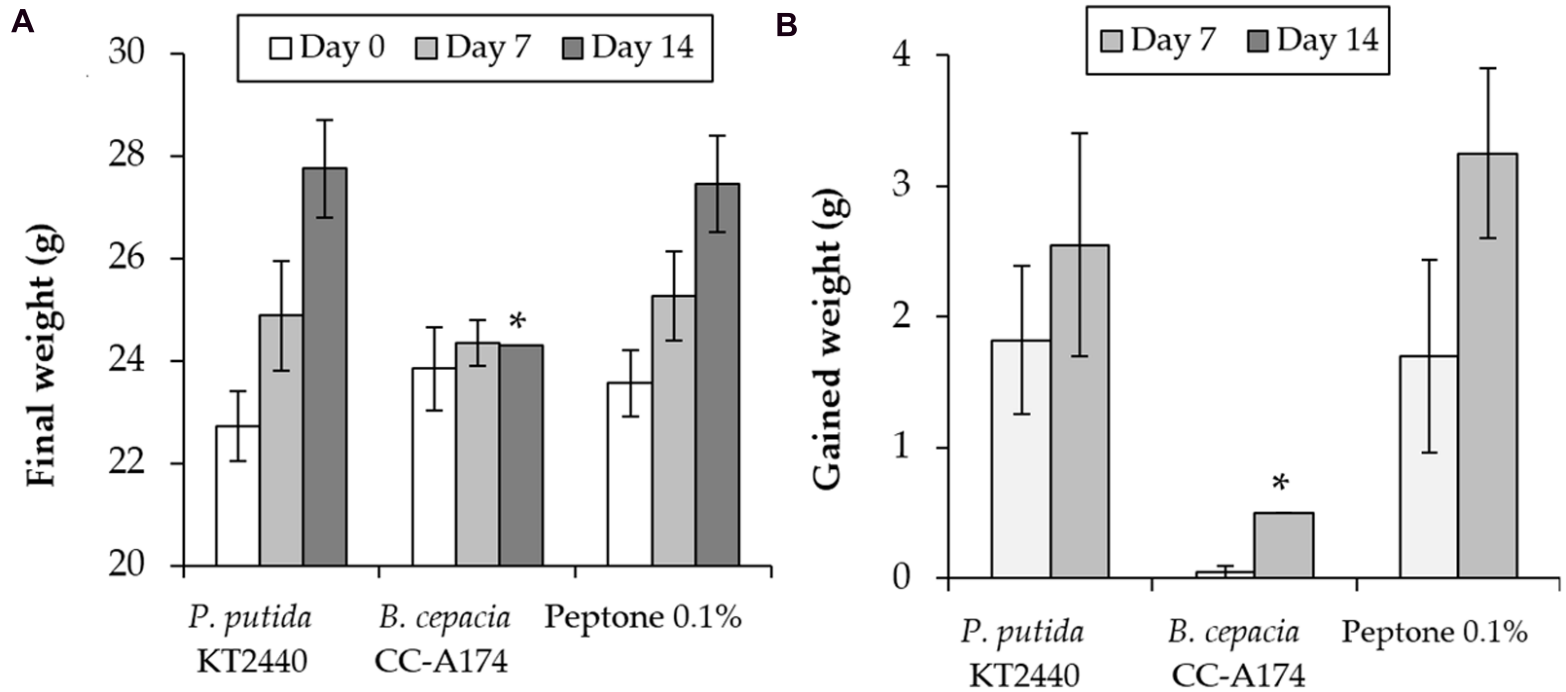
**Pathogenicity tests in laboratory mice (*Mus musculus* CD1).** Time course of changes in total weight **(A)** and weight gain **(B)** in mice after i.p. injection of *P. putida* KT2440, *B. cepacia* CC-A174 or a 0.1% peptone solution. Significant differences are indicated with an asterisk. The values shown are the mean and standard deviation of three measurements.

The laboratory mouse (*Mus musculus* CD1) is a reference animal in experimental models for human safety and as a model for small mammals and other vertebrate organisms in ecosystems in which agricultural products or biofertilizers may be used. We therefore wished to determine whether the results of laboratory tests in CD1 laboratory mice were consistent with the results of the tests described above. This was done to obviate the need for additional tests in vertebrates and to verify that the PGPB candidates tested here are safe for human health and environmentally friendly. We inoculated mice with both strains *P. putida* KT2440 and *B. cepacia* CC-A174, and compared weight gain and mortality rates in these mice and in a control group of animals that were injected with 0.1% peptone as a negative control. As is represented in **Figure 5**, all mice inoculated with *P. putida* KT2440 survived, weight gain was similar to that in the control group (mean initial weight 23 ± 0.5 g, mean final weight 26 ± 0.6 g). However, only 3 of the 5 mice inoculated with *B. cepacia* CC-A174 survived the observation period, and weight gain was almost null (mean initial weight 23.8 ± 0.8, mean final weight 24.3 ± 0.1 g).

### EHSI Model

Although models of pathogenesis based on the interaction between bacterial pathogens and higher organisms such as *C. elegans*, *E. foetida*, *C. carnea* or *A. bipunctata* have been used previously (Ruiz-Díez et al., 2003), to our knowledge there have been no attempts to integrate the results of different experimental tests to derive a single index. Probit function has been used to quantify the virulence of pathogenic bacteria such as *P. aeruginosa* PA14 in *C. elegans*. Mortality, weight and development time of *C. elegans* are parameters that have been used in one model of pathogenesis in humans (Adesemoye et al., 2008). On the basis of different ecotoxicological tests used to date (Schloter et al., 2003; Desneux et al., 2007), we included the bacterial community, nematodes and arthropods in our array of tests to obtain information on ecological and soil quality indicators. Any lethal or detrimental effect on bacterial metabolism can be interpreted as potentially harmful for soil bacteria and for the human microbiome, and consequently for human health (Gilbert et al., 2013; David et al., 2014; Heintz and Mair, 2014). This makes it important to investigate the effects of PGPB candidates on *C. elegans* and microbial metabolism and viability – an approach that considers both the environmental and human health (**Table 1**).

**Table 1:** Environmental and human safety index (EHSI) tests included and mouse pathogenicity tests with results of *Pseudomonas putida* KT2440 and *Burkholderia cepacia* CC-A174.

				Maximum Possible Value (MPV)	Score for test strain		Modeling for EHSI Categories
*Bioassay*	*Parameter*	*Observations*	*Target area*		*P. putida* KT2440	*B. cepacia* CC-A174	
**Environmental and human safety index**							
Sensitivity test with *E. coli* MC4100	CFUs/mL	A reduction of 50% indicates possible influence on soil microbiota	Soil microbiota	10	10	5	Mortality
Microtox^®^ Test (*V. fischeri*)	EC50	Genus *Vibrio* closely related to aquatic biology	Freshwater organisms	5	5	1.25	Development
		Effects extrapolated to microbiota	Soil microbiota				
Bioassay with *C. elegans*	No. Adults	Nematofauna are good markers of the status of soil microbiota	Soil microbiota	6	6	1.5	Reproduction
	No. Juveniles	Nematofauna are good markers of the status of soil organisms	Soil surface/Underground organisms	10,5	10,5	2.625	Reproduction
	No. Eggs	*Caenorhabditis elegans* is used as a model of pathogenicity in humans	Human health	6	6	1.5	Reproduction
	No. Deaths			25	25	6.25	Mortality
Bioassay with *C. carnea*	Length	Good markers of transfer in the food chain and sensitive biological control organisms	Soil surface organisms	1	1	1	Development
	Weight		Beneficial organisms	2	2	2	Development
	No. Deaths			3.75	3.75	3.75	Mortality
Bioassay with *A. bipucntata*	Length	Good markers of transfer in the food chain and sensitive biological control organisms	Soil surface organisms	1	1	1	Development
	Weight		Beneficial organisms	2	2	2	Development
	No. Deaths			3,75	3.75	3.75	Mortality
Bioassay/Ecotoxicity test with *E. foetida*	Length	Earthworms are good markers of the status of soil organisms since they are involved in the health of soil microbiota and soil ecosystems: nutrients, structure	Soil surface/Underground organisms	2	2	2	Development
	Weight			3	3	3	Development
	No. Juveniles		Soil microbiota	4,5	4.5	4.5	Reproduction
	No. Ootheca		Beneficial organisms	3	3	3	Reproduction
DaphtoxKit Test (*D. magna*)	EC50	Organisms greatly affected by changes in their environment	Freshwater organisms	7,5	5.625	1.875	Mortality
Test of bacterial effects on plants (based on pepper, *Capsicum annuum*)	Shoot length	The plant-microorganism balance	Soil microbiota	1	1	1	Development
	Dry weight (DW)	Agricultural species can carry pathogens that affect farm animals and humans (as habitat and food)	Human mealth	2	2	2	Development
	Relative water content (RWC)		Surface organisms	1	1	1	Development
**Final Score**				100	98.125	50	Maximum Score = 100
**Mouse pathogenicity**							
Bioassay with CD1 laboratory mice (*Mus musculus*)	Final weight	Mice are good markers of transfer in the food chain	Surface organisms	15	15	15	Development
	Weight gain			35	35	8.75	Development
	No. Deaths	Model of pathogenicity in humans	Human health	50	50	25	Mortality
**Final Score**				100	100	48.75	Maximum Score = 100

*Target areas covered by each test and by the alternative score based on biosafety tests in mice. The table shows the results used to obtain the total EHSI score for both test strains (A) and the score in the mouse pathogenicity model (B).*

**FIGURE 6:**
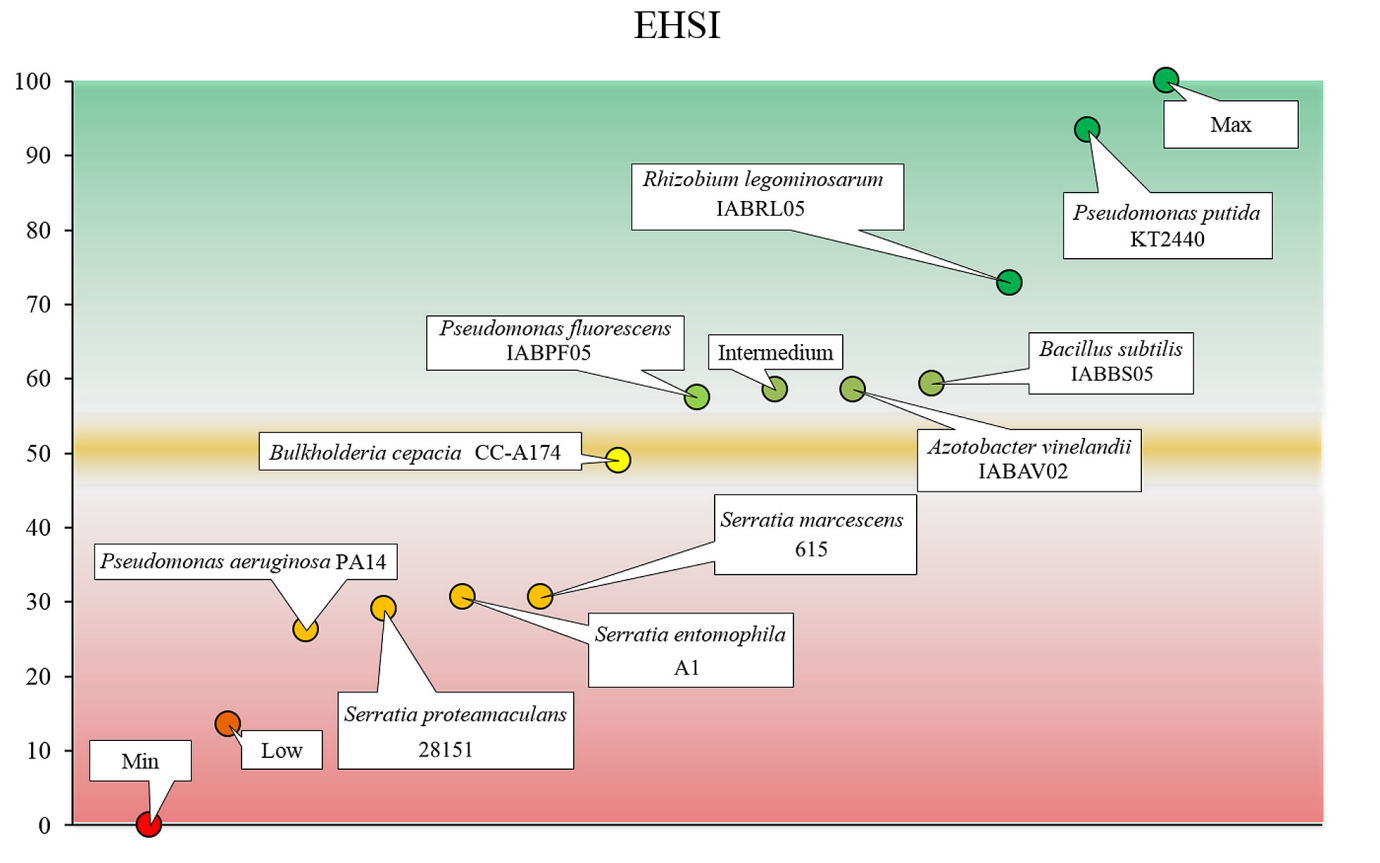
**Environmental and human safety index (EHSI).** Scores in the green zone indicate that the strain can be considered safe for use as a PGPB. Scores in the red zone indicate that additional tests should be done before the strain can be considered safe for use as a PGPB. The yellow area is considered a transition region of uncertainty. The values shown are the mean and standard deviation of three measurements.

We used the Delphi method to integrate the results from our panel of tests into a single value (Linstone and Turoff, 1975). Our ultimate aim was to develop a range of values that indicate whether a candidate PGPB strain is safe for human health and the environment (**Figure 6**). Values below a certain cutoff score (50 ± 0.5) indicate the need for additional safety tests before the candidate can be considered safe for use as a PGPB. We have attempted to develop a simple but accurate, rigorous and relevant set of tests to help decision-makers evaluate the safety of potential PGPB before approving the use of the candidate organism. We also aimed to determine whether the results of tests in mice were consistent with the results of the rest of the tests included in the panel as a way to minimize the use of vertebrate animals, as recommended by most animal ethics committees.

We termed the scale of values the *environmental and human safety index* (EHSI), which is scored from 0 to 100. Higher values indicate a greater likelihood that the bacterial strain of interest will be safe for use as a PGPB (**Figure 6**). The EHSI is based on tests of mortality (M), reproduction (R), and development (D) of target organisms. Mortality is the main factor used to determine the pathogenicity of a bacterial strain (Navas et al., 2007). However, the effects of the strain on reproduction can have a considerable influence on future populations of target organisms (Adesemoye et al., 2008). Developmental parameters of the target organism provide information about their ability to fulfill their intended environmental role (e.g., the role of some arthropods in pollination or pest control) as well as to reproduce (Desneux et al., 2007). On the basis of earlier research we assigned a given specific weight to each factor according to the following relative weight, where mortality represented 50%, reproduction represented 30% and development represented 20% of the final EHSI value. As shown in **Table 1**, the maximum possible sum of the values for the individual assays used to test mortality (M), reproduction (R), and development (D) is 100; hence the highest possible EHSI score is 100.

Maximum scores for different assays were weighted depending on the importance of each test and its relevance for human and environmental safety. For example, the highest possible score for *C. elegans* mortality was 50 (mortality equal to that observed with the non-pathogenic strain *E. coli* OP50, i.e., 0.6 deaths). This high score reflects the ecological importance of these nematodes as soil organisms and the importance of *C. elegans* as a model for pathogenesis in humans. Similarly, the importance of microorganisms in soil formation and their role as part of the human microbiome prompted us to assign a maximum score of 20 when the candidate PGPB has no effect on *E. coli* MC4100 viability. The effect on beneficial arthropods was scored to a maximum of 15 if no significant mortality was found in assays with *C. carnea* and *A. bipunctata* after the addition of the bacteria. The maximum score for the effect of the PGPB on aquatic fauna was 15 if no effect was seen on the EC50 of *D. magna*. In general, although the bacteria can alter the weight, size and fertility of *E. foetida*, they do not increase mortality thanks to the worm’s cellular and humoral defenses, even though the worm feeds on bacteria and they occupy its coelomic fluid. Consequently, the pathogenicity of the bacterial strain does not always reflect the mortality caused by the PGPB candidate; therefore the contribution of this assay to the EHSI score is low. We used analogous reasoning to establish weightings and maximum values for mortality, reproduction and development parameters for other organisms (**Table 1**) depending on the strength of their effect.

The score for the development parameter (D) was based on length and weight results obtained in *C. carnea, A. bipunctata*, and *E. foetida*, the EC50 from the Microtox test, and the results of DW and relative water content (RWC) in a plant model (*C. annuum*). This approach was used to ensure that the candidate PGPB had no effect on soil flora and thus complied with current EU legislation (which requires microorganisms added to the soil to have clear benefits for plant production). Our proposed a maximum score of 20 for this parameter: 5 for an increase in or no effect on plant length, 10 for an increase in plant weight, and 5 for an increase in (or no decrease in) RWC after exposure to the candidate PGPB.

To calculate the score for the reproduction parameter (R), we used the number of eggs, juveniles and adults from *C. elegans* and the number of ootheca and neonates from *E. foetida*. These data provide a good indication of how the PGPB affects changes in the demographics of soil organisms. Our approach included tests proposed by the Organization for Economic Co-operation and Development (OECD) to avoid the risk of biodiversity loss in soils due to the use of biocides, toxic agents or other harmful agents.

A PGPB candidate that does not alter any of the prespecified values used as safety indicators would obtain the maximum score of 100 and therefore would be considered safe to use under the assayed conditions. Intermediate scores vary depending on the magnitudes of the effects in different assays. If the difference compared to the negative control scores (i.e., the maximum score) is very small or negligible, and falls within the first quartile Q1 (0–24%), the final score is calculated by multiplying by 1.0. For example, if *C. elegans* mortality is same with the PGPB candidate and negative control (the non-pathogenic strain *E. coli* OP50), or if the difference is no greater than 24%, then the assigned score of 50 is multiplied by 1.0. If the difference compared to the negative control is low and falls within the second quartile Q2 (25–49%), then the score is multiplied by 0.75. If the difference is moderate and within the third quartile Q3 (50–74%), the score is multiplied by 0.5. If the difference is large and falls within the fourth quartile (75–95%), the score is multiplied by 0.25. If survival is extremely low (between 0 and 4%) with the PGPB candidate versus 100% with the negative control, the score is multiplied by 0.0.

To validate the potential applicability of this scoring system we calculated the EHSI for *P. putida* KT2440 and *B. cepacia* CC-A174 and contrasted the values with the results of viability tests in CD1 lab mice (*M. musculus*). As a result we assigned an EHSI score of 98.125 (innocuous) to *P. putida* KT2440 but a much lower score of 50 (further tests needed) to *B. cepacia* CC-A174 showing a clear correlation with results from animal tests. Pilot studies with twelve other PGPB candidates yielded a similar degree of consistency between EHSI scores and the results of animal tests (data not shown). On the basis of the criteria explained in this report, *P. putida* KT2440 can be considered safe to use as a PGPB, whereas additional tests of *B. cepacia* CC-A174 are needed to rule out potential risks to health and the environment posed by this strain, a risk group 2 microorganism that has been proposed as a PGPB. To evaluate the potential of the EHSI to reduce the number vertebrate animals needed for testing, we compared these results in terms of survival, weight gain and growth in CD1 laboratory mice after the intraperitoneal injection of bacteria, and found similar results with both strains. EHSI was calculated for *Rhizobium legominosarum* IABRL05, *Pseudomonas fluorescens* IABPF05, *Bacillus subtilis* IABBS05, and *Azotobacter vinelandii* IABAV02, *Serratia marcescens* 615 (Almaghrabi et al., 2013), *Serratia entomophila* A1 (Johnson et al., 2001), *Serratia proteamaculans* 28151 (Bai et al., 2002) and *P. aeruginosa* PA14 as a reference and their EHSI values calculation and representation among the *P. putida* KT2440 and *B. cepacia* CC-A174 controls can be found in **Table 2** and **Figure 6**.

**Table 2:** Environmental and human safety index (EHSI) tests included and mouse pathogenicity tests.

Environmental and human safety index		Score for test strain. Modeling for EHSI Categories							
*Bioassay*	*Parameter*	*S. marcescens* 615	*S. proteamaculans* 28151	*S. entomophila* A1	*P. aeruginosa* P14	*P. fluorescens* IABPF05	*A. vinelandii* IABAV02	*R. legominosarum* IABRL05	*B. subtilis* IABBS05
Sensitivityy test with *E. coli* MC4100	CFUs/mL	5	5	5	2.5	10	10	10	10
Microtox^®^ Test (*V. fischeri*)	EC50	1.25	1.25	1.25	1.25	2.5	2.5	2.5	2.5
Bioassay with *C. elegans*	No. Adults	1.5	1.5	1.5	1.5	3	3	3	3
	No. Juveniles	2.625	2.625	2.625	2.625	5.25	5.25	7.875	5.25
	No. Eggs	1.5	1.5	1.5	1.5	3	3	4.5	3
	No. Deaths	0	0	0	0	6.25	6.25	12.25	6.25
Bioassay with *C. carnea*	Length	0.75	1	1	1	1	0.75	1	1
	Weight	1.5	1.5	1	1	2	1.5	2	2
	No. Deaths	0	0	0	0	2.8125	2.8125	3.75	2.8125
Bioassay with *A. bipucntata*	Length	0.5	0.5	0.5	0.5	0.75	0.75	0.75	0.75
	Weight	0.25	0.25	0.25	0.25	1	1.5	2	1.5
	No. Deaths	0	0	0	0	1.875	2.8125	3.75	2.8125
Bioassay/Ecotoxicity test with *E. foetida*	Length	2	2	2	2	2	2	2	2
	Weight	3	3	3	3	3	3	3	3
	No. Juveniles	3.375	3.375	2.25	2.25	2.25	3.375	4.5	3.375
	No. Ootheca	2.25	2.25	2.25	1.125	3	2.25	2.25	2.25
DaphtoxKit^®^ Test (*D. magna*)	EC50	1.875	1.875	1.875	1.875	3.75	3.75	3.75	3.75
Test of bacterial effects on plants (based on pepper, *Capsicum annuum*)	Shoot length	1	1	1	1	1	1	1	1
	Dry weight	2	2	2	2	2	2	2	2
	RWC	1	1	1	1	1	1	1	1
**Final Score**		31.375	31.625	30	26.375	57.4375	58.5	72.875	59.25

*Target areas covered by each test. The table shows the results used to obtain the total EHSI score for the tested strains.*

## Discussion

Bacteria that will be released into the environment to promote plant growth should be safe for humans, animals and the environment. At present, however, there are no internationally harmonized, reliable protocols to evaluate the safety of these bacterial strains. We propose a panel of tests and an evaluation system to accurately determine the safety of bacterial strains. Our set of assays holds the potential to reduce the number of vertebrate animals needed for biosafety testing. We hope that these tests will help policy makers in their efforts to develop new regulations. The EHSI is a new instrument that holds the potential to facilitate the prediction of potential harms to human health and the environmental caused by organisms that are under investigation for use as PGPB. The combination of tests in microorganisms and pathogenicity assays in laboratory mice can help reduce the need to use vertebrates in experimental research – one of the aims of current 3Rs policies (reduce, replace, refine) regulating the use of animals for research purposes. The modular nature of the EHSI makes it easy to interchange target organisms depending on the local environmental. For example, if a reduction is observed in the local bee population and no river or fresh water habitats are located near target crops for a particular PGPB, assays in *D. magna* could be replaced with assays in bees. We therefore view EHSI as a tool that can be adapted to local priorities and policies. In addition, this index can be used to evaluate the potential risks associated with microorganisms that might require release for other biotechnological applications. For example, both bacterial strains tested here, *P. putida* KT2440 and *Burkholderia cepacia* CC-Al74, have been proposed for release for bioremediation in polluted soils, so EHSI could be used to test their probable impact before release. The index is easily adaptable by opting to evaluate, for example, the efficiency of the candidate organism in its ability to biodegrade a soil pollutant instead of evaluating its plant-growth promoting effect. Additional studies with well known PGPB validate the value of this index, however, additional assays with other bacteria can refine the model for future applications of the EHSI and to carry out further quality assessments of this tool.

## Author Contributions

JV and SA have performed the experimental assays. JV, JG-L, and AN have performed the statistical analysis. JV and MM have designed and written the article.

## Conflict of Interest Statement

The authors declare that the research was conducted in the absence of any commercial or financial relationships that could be construed as a potential conflict of interest.
